# Association between apolipoprotein E gene polymorphism and the risk of coronary artery disease in Hakka postmenopausal women in southern China

**DOI:** 10.1186/s12944-020-01323-6

**Published:** 2020-06-16

**Authors:** Jingyuan Hou, Qiaoting Deng, Xuemin Guo, Xunwei Deng, Wei Zhong, Zhixiong Zhong

**Affiliations:** 1 Clinical Core Laboratory, Meizhou People’s Hospital (Huangtang Hospital), Meizhou Hospital Affiliated to Sun Yat-sen University, Meizhou, 514031 People’s Republic of China; 2 Guangdong Provincial Engineering and Technology Research Center for Molecular Diagnostics of Cardiovascular Diseases, Meizhou, 514031 People’s Republic of China; 3 Guangdong Provincial Key Laboratory of Precision Medicine and Clinical Translational Research of Hakka Population, Meizhou, 514031 People’s Republic of China; 4 Center for Cardiovascular Diseases, Meizhou People’s Hospital (Huangtang Hospital), Meizhou Hospital Affiliated to Sun Yat-sen University, No. 63 Huangtang Road, Meijiang District, Meizhou, 514031 People’s Republic of China

**Keywords:** Coronary artery disease, Apolipoprotein E, Gene polymorphism, Postmenopausal, Hakka

## Abstract

**Background:**

Apolipoprotein E (*APOE*) is involved in the pathogenesis of atherosclerosis and conveys a higher risk of coronary artery disease (CAD). The aim of the present study was to investigate the possible association between *APOE* gene polymorphism and the risk of CAD in postmenopausal Hakka women in southern China.

**Methods:**

The *APOE* genotypes of 653 CAD patients and 646 control participants were determined by the polymerase chain reaction (PCR) and hybridization to a Sinochip.

**Results:**

The prevalence of each *APOE* genotype differed between CAD patients and control participants (*P* = 0.011). The *E3/E3* genotype was the most common and the *E2/E2* genotype was the least common in the study sample. Moreover, the presence of *ε4* allele was associated with higher serum concentrations of triglycerides (TG), total cholesterol (TC) and low-density lipoprotein-cholesterol (LDL-C), and lower concentration of high-density lipoprotein-cholesterol (HDL-C). Multiple logistic regression analysis revealed that participants with *ε4* allele have a significantly higher risk of CAD after adjustment for the presence of diabetes mellitus and hypertension, and their serum uric acid, TC, and LDL-C concentrations (adjusted odds ratio (OR) 1.50, 95% confidence interval (CI) 1.10–2.05, *P* = 0.010).

**Conclusions:**

The present results suggest that *APOE* polymorphism is associated with a higher risk of CAD in postmenopausal Hakka women in southern China.

## Background

Coronary artery disease (CAD) remains one of the most complex diseases with a high morbidity and mortality worldwide [1, 2]. In spite of significant improvements in the clinical management of CAD, the pathogenesis of atherosclerosis, which underlies the CAD, remains to be fully characterized. It is well established that both genetic and environmental risk factors are involved in the development of CAD [3, 4]. Factors including advanced age, hypertension, diabetes mellitus, smoking, and poor diet have been shown to increase the risk of CAD [5, 6]. In particular, dyslipidemia is a significant contributor for the progression of atherosclerotic lesions, and this may result from variations of or epigenetic modifications to genes involved in lipid metabolism [7–9]. Therefore, there has been a great deal of interest in the effect of genetic variants on the risk of CAD.

Apolipoprotein E (APOE) is a glycoprotein that consists of 299 amino acids and is encoded by a gene located on chromosome 19 at position q13.2 [10]. There are three common alleles at the *APOE* locus, namely *ε2*, *ε3*, and *ε4*, which yield six possible genotypes: *E2/E2*, *E2/E3*, *E3/E3*, *E3/E4*, *E4/E4*, and *E2/E4* [11]. The polymorphisms of *APOE* have been reported to be associated with the regulation and metabolism of lipids [12]. The most common isoform is E3, which facilitates the scavenging of certain lipoproteins from the circulation, whereas the E2 and E4 isoforms have different affinities for the low-density lipoprotein (LDL) receptor, thereby affecting circulating lipid concentrations [13].

In the past few decades, the crucial role of *APOE* in the pathogenesis of atherosclerosis has been recognized [14, 15]. More recently, several studies have investigated the relationships between *APOE* polymorphisms and cardiovascular and cerebrovascular disease [16–18]. However, the identified associations between *APOE* polymorphisms and CAD were highly inconsistent [19]. Furthermore, no information has been published regarding the relationship between *APOE* polymorphism and the risk of CAD in the Hakka ethnic group in China. Therefore, the aim of the present study was to investigate the possible association between *APOE* gene polymorphism and the risk of CAD in postmenopausal Hakka women in southern China.

## Methods

### Study participants

A total of 1299 postmenopausal women were recruited from the inpatient service of Meizhou People’s Hospital (Huangtang Hospital) between May 2016 and August 2018 (653 women with confirmed CAD and 646 women without CAD, who acted as controls). The enrolled women were aged over 50 years (66.87 ± 10.09 years, *n* = 1299) and self-reported to have been in menopause for at least 12 months. CAD was defined as stenosis > 50% in at least one segment of a major coronary artery (the left main coronary trunk, anterior descending branch, left circumflex artery and/or right coronary artery). The control participants did not have lumen stenosis on coronary angiography or evidence of cardiovascular disease on physical examination. Coronary angiograms were interpreted by two experienced cardiologists who did not have knowledge of the patients’ clinical history. Hypertension was defined as a mean of 3 independent measures of blood pressure ≥ 140/90 mmHg or currently receiving hypertension treatment. Diabetes mellitus was defined as a fasting glucose levels ≥126 mg/dL, or non-fasting glucose levels ≥200 mg/dL, or current treatment with oral hypoglycemic agents or insulin. Hyperhomocysteinemia was defined as a serum homocysteine concentration > 15 μmol/L. Hyperuricemia was defined as uric acid (UA) ≥ 420 mmol/L in men or ≥ 360 mmol/L in women. Hyperlipidemia was defined as level of total cholesterol (TC) > 5.5 mmol/L, triglycerides (TG) > 1.7 mmol/L, LDL-cholesterol (LDL-C) > 3.4 mmol/L, high-density lipoprotein-cholesterol (HDL-C) < 1.0 mmol/L. The exclusion criteria were congenital or valvular heart disease, severe renal or hepatic disease, thyroid dysfunction, autoimmune disease, or malignant disease, or use of lipid-controlling drugs.

The study was approved by the Institutional Review Boards at Meizhou People’s Hospital (Huangtang Hospital) and conducted in compliance with the ethical guidelines of the 1975 Declaration of Helsinki. Signed consent form was obtained from each participant before their enrollment in the study. All the participants lived in the same region and were confirmed to be of Hakka origin by consideration of the ethnic origin of their parents and grandparents.

### DNA extraction and genotyping

A 4-mL venous blood sample was drawn from each participant into an EDTA sample tube, then extraction of genomic DNA from peripheral blood mononuclear cells was performed using a QIAamp DNA blood kit (Qiagen, Hilden, Germany). The quality and quantity of the DNA were evaluated using a Nano-Drop 2000™ spectrophotometer (ThermoFisher Scientific, Waltham, MA, USA). Genotyping of the *APOE* gene single nucleotide polymorphisms (rs429358 and rs7412) were performed with a commercially available kit (Zhuhai Sinochips Bioscience Co., Ltd., Guangdong, China). The polymerase chain reaction (PCR) parameters were as follows: 2 min at 50 °C; 15 min at 95 °C; 45 cycles of denaturation at 94 °C for 30 s; and annealing and extension at 65 °C for 45 s. After PCR amplification, the PCR products were subsequently added to the gene chip (*APOE* genotype test kit) for hybridization. Finally, a gene chip scanner was used to interpret the data. Five percent of the samples were also selected randomly for sequencing to confirm the results of the genotyping and the concordance rate was 100%.

### Biochemical measurements

Fasting blood samples were drawn from all participants and centrifuged at 3000×*g* for 10 min, and aliquots were stored at − 80 °C before analysis. Serum concentrations of TG, TC, LDL-C, HDL-C, and UA were measured strictly according to the standard methods in the hospital clinic laboratory.

### Statistical analysis

All statistical analysis were performed using SPSS version 19.0 (IBM Inc., Armonk, NY, USA). Continuous variables were expressed as means ± standard deviations and were analyzed with the Student’s *t*-test or ANOVA. Categorical variables were expressed as numbers and percentages and were analyzed with the Chi-square test. Hardy–Weinberg equilibrium in the CAD patients and controls was evaluated by Chi-square test*.* Odds ratio (OR) and 95% confidence interval (CI) were calculated to express the relative risk of disease, using SPSS logistic regression. *P* < 0.05 was considered to represent statistical significance.

## Results

### Baseline clinical characteristics of the study participants

The baseline clinical characteristics of all the postmenopausal participants in the study are summarized in Table 1. The study sample consisted of 1299 postmenopausal women (mean age 68.16 ± 9.42 years for the 653 angiographically confirmed CAD patients and 65.57 ± 10.57 years for the 646 control participants). Notably, the age, blood pressure, and the prevalences of hypertension, diabetes mellitus, hyperhomocysteinemia, hyperlipidemia, and hyperuricemia were significant differences between the two groups (all *P <* 0.05). The CAD patients had significantly higher serum concentrations of UA, TC, and LDL-C than the control participants (all *P <* 0.05), but there were no significant differences in the TG and HDL-C concentrations between the two groups (all *P >* 0.05).

**Table 1:** Baseline clinical characteristics of the study participants

Characteristics	CAD patients	Controls	*P* value
	(*n* = 653)	(*n* = 646)	
Age (years)	68.16 ± 9.42	65.57 ± 10.57	< 0.001
SBP (mm Hg)	141.06 ± 22.78	127.07 ± 15.37	< 0.001
DBP (mm Hg)	81.44 ± 13.96	79.74 ± 12.78	0.020
Hypertension (%)	441 (67.53)	290 (44.89)	< 0.001
Diabetes mellitus (%)	223 (34.15)	139 (21.55)	< 0.001
Hyperhomocysteinemia (%)	83 (12.71)	19 (2.94)	< 0.001
Hyperuricemia (%)	80 (12.25)	16 (2.48)	< 0.001
Hyperlipidemia (%)	238 (36.45)	113 (17.49)	< 0.001
Uric Acid (μmol/L)	349.66 ± 113.79	290.77 ± 116.53	< 0.001
TG (mmol/L)	2.08 ± 1.74	1.89 ± 1.22	0.080
TC (mmol/L)	5.39 ± 1.23	4.97 ± 1.39	< 0.001
LDL-C (mmol/L)	3.05 ± 0.88	2.80 ± 0.89	< 0.001
HDL-C (mmol/L)	1.26 ± 0.33	1.28 ± 0.42	0.330

*CAD* Coronary artery disease, *SBP* Systolic blood pressure, *DBP* Diastolic blood pressure, *UA* Uric acid, *TG* Total triglycerides, *TC* Total cholesterol, *LDL-C* Low density lipoprotein cholesterol, *HDL-C* High density lipoprotein cholesterol

### The distributions of genotypes and alleles of the *APOE* gene in the CAD patients and control participants

The distributions of genotypes and alleles of the *APOE* gene in the CAD patients and control participants are summarized in Table 2. The genotype distributions in both the CAD patients and control participants were consistent with Hardy-Weinberg equilibrium (*χ*^2^ = 1.79, *P* = 0.77 and *χ*^2^ = 2.06, *P* = 0.73, respectively). The distributions of *APOE* genotypes and alleles significantly differed between the two groups (*P* = 0.011 and *P* = 0.003, respectively). The *E3/E3* genotype was the most common in both groups (67.69% of CAD patients and 69.35% of control participants), followed by the *E3/E4* genotype (19.14% of CAD patients and 14.24% of control participants), and the *E2/E3* genotype (10.11% of CAD patients and 14.09% of control participants).

**Table 2:** The distributions of genotypes and alleles of the *APOE* gene in the CAD patients and control participants

*APOE*	CAD patients	Controls	*P* value
	(*n* = 653)	(*n* = 646)	
Genotype			
*E2/E2*	3 (0.46)	2 (0.31)	
*E2/E3*	66 (10.11)	91 (14.09)	
*E3/E3*	442 (67.69)	448 (69.35)	
*E3/E4*	125 (19.14)	92 (14.24)	
*E2/E4*	6 (0.92)	10 (1.55)	0.011
*E4/E4*	11 (1.68)	3 (0.46)	
*HWE*	χ^2^ = 1.79, *P* = 0.77	χ^2^ = 2.06, *P* = 0.73	
Allele			
*ε2*	78 (5.97)	105 (8.13)	
*ε3*	1075 (82.31)	1079 (83.51)	0.003
*ε4*	153 (11.72)	108 (8.46)	

*HWE* Hardy-Weinberg equilibrium

The participants were then allocated to three subgroups: *ε2* carriers, which included individuals with the *E2/E2* or *E2/E3* genotypes, *ε3* carriers, which included individuals with the *E3/E3* genotype, and *ε4* carriers, which included individuals with the *E3/E4* or *E4/E4* genotypes. Allele *ε3* was the most common (82.31% of CAD patients and 83.51% of control participants), followed by allele *ε4* (11.72% of CAD patients and 8.46% of control participants), and allele *ε2* (5.97% of CAD patients and 8.13% of control participants). The allele frequency of *ε4* was significantly higher in CAD patients than in the control participants (*P* = 0.003).

### Relationships between serum lipid profile and *APOE* allele in CAD patients and control participants

The relationships between allelic carrier status (*ε2*, *ε3*, and *ε4* groups) and serum lipid profile are summarized in Table 3. The *APOE ε2* and *ε4* alleles were considered to play opposing roles in lipid metabolism and the incidence of CAD, therefore, participants with the *E2/E4* genotype (*n* = 16) were excluded. As expected, the serum TG, TC, HDL-C, and LDL-C concentrations significantly differed among the *ε2*, *ε3*, and *ε4* groups of CAD patients. Specifically, the *ε4* carriers had significantly higher concentrations of TG, TC, and LDL-C, and lower concentration of HDL-C than the other groups, while the *ε2* carriers showed the opposite results. Additionally, the TC and LDL-C concentrations of the control participants showed similar trends to those in the CAD group. However, there were no significant impacts of the *APOE* polymorphism on the TG and HDL-C concentrations in the control participants.

**Table 3:** Relationships between serum lipid profile and *APOE* allele in CAD patients and control participants

Lipid level	CAD patients				Controls			
(mmol/L)	*ε2* (*n* = 69)	*ε3* (*n* = 442)	*ε4* (*n* = 131)	*P* value	*ε2* (*n* = 93)	*ε3* (*n* = 448)	*ε4* (*n* = 102)	*P* value
TG	1.87 ± 1.20^△^	1.96 ± 1.35^△^	2.57 ± 2.74^*^	0.001	1.77 ± 1.39	1.91 ± 2.51	1.94 ± 1.28	0.846
TC	5.04 ± 1.09^*△^	5.41 ± 1.16	5.52 ± 1.48	0.024	4.69 ± 1.29^△^	4.95 ± 1.38^△^	5.26 ± 1.47^*^	0.017
LDL-C	2.56 ± 0.75^*△^	3.09 ± 0.85	3.20 ± 0.95	< 0.001	2.60 ± 0.93^△^	2.77 ± 0.84^△^	3.07 ± 0.99^*^	0.001
HDL-C	1.29 ± 0.37^△^	1.28 ± 0.34^△^	1.18 ± 0.27^*^	0.009	1.30 ± 0.39	1.28 ± 0.44	1.23 ± 0.38	0.416

*P* value shows the differences compared between groups (*ε2*, *ε3*, *ε4*)

*P < 0.05 versus corresponding *ε3* group

^△^*P* < 0.05 versus corresponding *ε4* group

### Logistic regression analysis of the risk of CAD in the Hakka population

Logistic regression analysis was performed to determine independent predictors for CAD (Table 4). On univariate regression analysis, there were significantly higher risks of CAD in the presence of the *ε4* allele, diabetes mellitus, hypertension and high UA, TC, and LDL-C concentrations (all *P <* 0.05). Further multiple logistic regression analysis indicated that participants with *ε4* allele had a significantly higher risk of CAD after adjustment for the established risk factors (adjusted OR 1.50, 95% CI 1.10–2.05, *P* = 0.010).

**Table 4:** Logistic regression analysis of the risk of CAD in the Hakka population

Variables	Crude values			Adjusted values^a^		
	*β*	*P* value	OR (95% CI)	*β*	*P* value	OR (95% CI)
*ε4* carrier	0.422	0.004	1.53 (1.14–2.04)	0.406	0.010	1.50 (1.10–2.05)
Diabetes mellitus	0.637	< 0.001	1.89 (1.48–2.42)	0.493	< 0.001	1.64 (1.26–2.13)
Hypertension	0.942	< 0.001	2.57 (2.05–3.22)	0.819	< 0.001	2.27 (1.79–2.88)
UA	0.231	< 0.001	1.21 (1.14–1.36)	0.218	< 0.001	1.20 (1.13–1.36)
TC	0.255	< 0.001	1.29 (1.18–1.41)	0.283	< 0.001	1.33 (1.10–1.61)
LDL-C	0.326	< 0.001	1.39 (1.22–1.57)	0.249	< 0.001	1.28 (1.08–1.54)

*OR* Odds ratio, *CI* Confidence interval, *UA* Uric acid, *TC* Total cholesterol, *LDL-C* Low density lipoprotein cholesterol

^a^The accuracy of model for the risk of the CAD was 65.6%

## Discussion

CAD is a multifactorial disorder with high incidences of disability and mortality around the world [2]. The prevalence of CAD is rising dramatically in China, alongside changes in lifestyle and an increase in lifespan [20]. CAD is considered to result from an interaction between genetic and environmental factors [4]. Several studies have suggested that *APOE* variants increased the risk of developing CAD [21, 22]. It is noted that this was the first to identify an association between *APOE* polymorphisms and the risk of CAD in postmenopausal Hakka women in southern China. The present study revealed that plasma lipid concentrations were significantly affected by genetic variations at the *APOE* gene locus. Significantly higher serum TG, TC, and LDL-C concentrations and significantly lower serum HDL-C concentration were found in CAD patients than in control participants. Furthermore, a statistically significant association between the *ε4* allele and a higher risk of CAD has also been identified in the study sample. This association remained significant when adjusted for several important cardiovascular risk factors, such as the presence of diabetes mellitus or hypertension and the serum UA and TC concentrations, in multiple logistic regression analysis.

APOE is an important plasma protein and its synthesis, secretion and metabolism are mainly completed in the liver [12]. The *APOE* gene is polymorphic, with three possible alleles: *ε2*, *ε3*, and *ε4*, which encode the isoforms E2, E3, and E4. The prevalences of the *APOE* genotypes vary widely across geographical areas and ethnic groups [23]. In most populations, *E3/E3* is the most prevalent genotype and *ε3* is the commonest allele. *ε4* is relatively common in northern Europeans and African Americans, while Asians have low prevalences of *ε2* and *ε4* [14, 24]. The present study have explored the prevalences of *APOE* genotypes and alleles in postmenopausal CAD patients and controls. In the CAD patients, the prevalences of the *E2/E2*, *E2/E3*, *E3/E3*, *E3/E4*, *E2/E4* and *E4/E4* genotypes were 0.46, 10.11, 67.69, 19.14, 0.92, and 1.68%, respectively, and in the control participants they were 0.31, 14.09, 69.35, 14.24, 1.55, and 0.46%, respectively. Thus, the *E3/E3* genotype was the most common and the *E2/E2* genotype was the least common in this sample, which is in broad agreement with those for other populations [13, 25].

The influence of *APOE* polymorphisms on CAD can be largely attributed to its effects on blood lipid profile, as shown in a previous large prospective study [26]. The *APOE* gene is known to be a significant determinant of the human lipid profile. The *ε3* allele of *APOE* promotes the clearance of TG-rich lipoproteins, and therefore helps prevent atherosclerosis [11]. However, a previous study showed that the *ε4* allele was relevant to the elevated serum TC and LDL-C concentrations, and consequently greater risks of atherosclerosis and ischemic heart disease [15]. A statistically significant association between the *APOE* allele and serum lipid concentrations have been confirmed in the present study. High serum TG, TC, and LDL-C concentrations were found in postmenopausal carriers of the *ε4* allele in the Hakka population. This connection between the *ε4* allele and cholesterol may be explained by stronger binding of lipid by *E4*, resulting from a single amino acid substitution (Cys112Arg) in *APOE* [27].

The associations between polymorphisms in the *APOE* gene and CAD identified in observational studies are still being debated. However, previous studies have suggested that the *ε4* allele was strongly associated with higher cardiovascular risk in several ethnic groups [15, 28]. Indeed, it has been reported that the *ε4* allele may serve as an independent genetic predictor of the severity of CAD in male Chinese patients [29]. In addition, another study demonstrated that diabetic carriers of the *APOE ε4* allele had an increased risk of CAD in western Iran [30]. In this study, logistic regression analysis showed that the *APOE ε4* allele independently increased the risk of CAD in postmenopausal women, which were consistent with the above findings.

However, other studies have found conflicting results. Erkki et al. showed that the *APOE ε4* allele was significantly associated with a higher risk of coronary atherosclerosis in men in early middle age, but not in older men [31]. On the contrary, Letonja et al. did not find such a relationship between the *APOE* phenotype and CAD risk in Caucasian women younger than 65 years [32]. Another study conducted in African-Americans and Caucasians failed to show a correlation between the *APOE* polymorphism and the risk of developing CAD, after adjustment for several conventional risk factors, such as age, sex, and the TG and HDL-C concentrations [33]. The reasons for these inconsistent results remain to be determined. However, it was speculated that these discrepancies may be explained by differences in sample size, patient selection, age, sex, lifestyle, and ethnicity, as well as by genotype-phenotype relationships and gene-environment interactions [34–36].

### Study strengths and limitations

There are several strengths of this study. It was the first time to investigate the potential association between *APOE* gene polymorphism and the presence of CAD in Hakka postmenopausal women in southern China. The study included the clinical characteristics, lipid profiles and *APOE* gene polymorphism indicators into the analysis to exclude the influence of related confounding factors on the results. Some potential limitations of this study also should be noted. First, selection bias may have existed, because the recruited control participants came from a population attending hospital. Second, the sample size of this study was insufficient, which might have under-powered the study. Thus, further studies with larger samples are warranted to confirm these findings. Third, because the study was conducted only in Hakka Chinese people, the findings cannot be readily generalized to other populations.

## Conclusions

In conclusion, the present findings suggest that *APOE* is a susceptibility locus for CAD in postmenopausal Hakka women in southern China. The *APOE ε4* allele was significantly associated with high serum lipid concentrations and was an independent risk factor for CAD, and this association remained significant after adjustment for multiple potential confounding factors. Therefore, *APOE* genotyping may be useful to identify individuals at high risk of CAD and provide guidance for the institution of individualized preventive strategies and therapies for patients.

## Data Availability

All data generated or analysed during this study are included in this published article.

## Abbreviations

*APOE*: Apolipoprotein E
CAD: Coronary artery disease
CI: Confidence intervals
DBP: Diastolic blood pressure
HDL-C: High-density lipoprotein cholesterol
LDL: Low-density lipoprotein
LDL-C: LDL cholesterol
OR: Odds ratio
PCR: Polymerase chain reaction
SBP: Systolic blood pressure
TG: Triglycerides
TC: Total cholesterol
UA: Uric acid
